# Who Has Done It? Exploring Gaze Agency in Obsessive-Compulsive Checkers

**DOI:** 10.3389/fnint.2017.00039

**Published:** 2017-12-22

**Authors:** Mattia Giuliani, Riccardo M. Martoni, Regina Gregori Grgič, Sofia A. Crespi, Maria C. Cavallini, Claudio de’Sperati

**Affiliations:** ^1^Department of Clinical Neurosciences, IRCCS San Raffele-Ville Turro, Milan, Italy; ^2^Laboratory of Action, Perception and Cognition, Faculty of Psychology, Vita-Salute San Raffaele University, Milan, Italy; ^3^Experimental Psychology Unit, Division of Neuroscience, IRCCS San Raffaele Scientific Institute, Milan, Italy; ^4^CERMAC, Department of Neuroradiology, IRCCS San Raffaele Scientific Institute, Milan, Italy

**Keywords:** agency, OCD, eye movements, checker, cognitive flexibility

## Abstract

The sense of agency (SoA) is a multifaceted construct, which can be defined as the ability to understand the causal relationships between our actions and sensory events. Obsessive-Compulsive Disorder (OCD) patients with checking compulsions often report a “lack of action completion” sensations, which has been conceptualized in the so-called “Not Just Right Experiences” construct. An intriguing explanation of this phenomenon comes from Belayachi and Van der Linden (2009, 2010), who suggest that OCD-checking patients are more prone to specify their action in a relatively molecular and inflexible way. Currently, there are no studies in literature which address this issue in OCD patients, except for the one of Gentsch et al. (2012), who suggested an altered SoA in these patients. Here we exploited a novel construct, gaze agency, to evaluate causal attribution capabilities in a group of 21 OCD patients (checkers) and matched healthy controls (HCs). Basically, two tasks targeted observers’ capability to identify their own eye movements as the cause of concurrently presented beeps, which allowed us to measure agency sensitivity as well as subtle agency alterations in an ecological setting. We found a poorer performance in OCD patients as compared to HCs in many parameters of our tasks, suggesting a difficulty with causal attribution possibly due to both a reduced cognitive flexibility and a less functional gaze agency in OCD patients.

## Introduction

One of the most basic aspects of self-awareness is the feeling of control and ownership on our actions: indeed, we normally perceive ourselves as the authors and executors of our intended acts (Haggard and Eitam, 2015). This feeling is known as “agentive self-awareness”, which comprises the sense of agency (or *SoA*), that is, the feeling to cause and to be in control of those actions (and their effects) that are coherent with our own intentions and goals (Haggard and Eitam, 2015).

SoA can be theoretically divided into two components: (1) *Agency feeling* (or *experience*), which can be defined as the “non-conceptual, automatic registration of whether I am the agent or not” (Synofzik et al., 2013) of an action, and it is based on sensory and premotor signals; (2) *Agency belief*, which can be defined as the “formation of a belief about who the initiator of the movement was” (Synofzik et al., 2013), and it is based on our judgment about the authorship of an action. Thus, SoA can be viewed as a sort of “phenomenal background” of our daily experience (Haggard and Eitam, 2015).

A function of SoA is the differentiation between self- and externally-generated actions and thoughts (Gallagher, 2000). For this reason, it is not surprising that impairments of SoA have been linked to many neurological and neuropsychiatric illnesses and phenomena, such as schizophrenia, psychosis and anarchic hand syndrome (Feinberg, 1978; Frith et al., 2000; de Vignemont and Fourneret, 2004; Jeannerod, 2009; Cantagallo et al., 2010; Synofzik and Voss, 2010; Maeda et al., 2013).

Furthermore, SoA has been included as one of the social processes subconstruct in the *Research Domain Criteria* (RDoC) matrix, a promising approach to create a new dimensional research framework for studying mental disorders. Indeed, being conceived as a transversal theoretical account, SoA could be impaired in other mental diseases beyond schizophrenia. Belayachi and Van der Linden (2009) hypothesized that also obsessive-compulsive (OC) patients with checking compulsions could show an altered SoA. These authors proposed that checking compulsions could be triggered by an incapability in reaching a sense of task completion, relating to both action and perception (the so-called “Not Just Right Experiences”; see Coles et al., 2003). As Belayachi and Van der Linden (2010) noted, theories on SoA offered sound theoretical context for understanding how “such a basic feeling of doing can be modulated by the unconscious perception of a correspondence between observed action effects and expected ones”. SoA is a new research domain in Obsessive-Compulsive Disorder (OCD): so far, only one study has investigated it in OCD patients (Gentsch et al., 2012). Gentsch et al. (2012) have hypothesized that OCD patients may have an altered motor system forward model, since several studies have connected OCD to functional abnormalities in brain regions involved in motor control and action monitoring (Greenberg et al., 2000; Ursu et al., 2003; Mantovani et al., 2006; Yücel et al., 2007). They operationalized this SoA abnormality as an altered sensory gating process as indexed by N1 event-related potentials (ERPs) component, based on the fact that a reduction of the N1 component reflects sensory suppression processes when self-generated auditory or visual events are compared to passively ones (Schafer and Marcus, 1973; Curio et al., 2000; Gentsch and Schütz-Bosbach, 2011). The aim of Gentsch’s study was to demonstrate that “gating of sensory information on the basis of motor predictions would be reduced in OCD, due to a dysfunctional system of motor control” (Gentsch et al., 2012).

Their results showed that OC patients exhibit a lack of N1 suppression during self-generated visual events (i.e., by a key press), linking this impairment to a deficient internal motor prediction capacity, which may in turn explain the aforementioned OCD patients’ incapability in reaching a sense of action completion and satisfaction. Interestingly, patients’ explicit judgments of agency did not differ from healthy controls (HCs) ones, thus suggesting that automatic mechanisms were at play. Nonetheless, Gentsch et al. (2012) showed a tendency to increased agency judgments, which seemed to be biased by “inflated beliefs of special personal responsibility”. Thus, as the authors stated, it is unclear how perceptual-motor mechanisms, on the one hand, and prior beliefs about personal responsibility, on the other hand, play a role in agency feelings alterations.

The previous studies refer to a widely used theoretical framework to study SoA: the comparator model (Frith et al., 2000). It basically claims that the brain computes SoA by predicting the sensory consequences of an action, generated by an efference copy (and its consequent corollary discharge) of the motor command, and comparing them with the current sensory outcomes (i.e., visual and proprioceptive) produced by that action. When these two kinds of information match, that is, when the predicted sensory feedback is in line with the actual one, the action is considered as self-caused, while a mismatch condition leads to an external attribution of causality. Interestingly, in 1987 Roger K. Pitman proposed the so-called Cybernetic Model (CM) of OCD, which assumes that any behavior (or action) can be represented as a comparative process between an internal reference signal computed in a forward model (i.e., an efference copy) and the sensory input resulting from the behavior/action (Pitman, 1987). Formally, the process described by Pitman is identical to SoA comparator model, and he sharply stated that, regardless of the symptomatology, the “core problem of OCD is the persistence of high error signals, or mismatch, that cannot be reduced to zero through behavioral output” (Pitman, 1987). This mismatch finds its phenomenological expression in the aforementioned pervasive “sense of incompleteness”, which leads to a repetitive and stereotyped execution of actions in order to reduce the error signals. However, despite the simplicity and refinement of the comparator model, it is not clear yet how it could account for OCD behavior.

In a recent opinion article, Thakkar et al. (2017) propose the eye as a reliable effector in studying SoA, as oculomotor neurophysiology is well-known also in terms of corollary discharge mechanisms. Recently, Gregori Grgič et al. (2016) suggested a new tool to assess “gaze agency”, defined as the “sense of gaze-operated self-agency in non-social context”, that is, the awareness to cause something in the environment through the movements of our eyes. Basically, their approach consisted of pairing a novel task for spontaneous agency discovery and a psychophysical task for explicit agency monitoring, both based on eye movements (for this kind of dual-task approach see also de’Sperati and Baud Bovy, 2017). It should be noted that in our daily life we do not use our eyes to physically modify the environment, although with gaze-operated devices this might soon become a widespread reality. It is thus important to study agency through such novel gaze capability, whose “naturalization” requires becoming aware of this very possibility and an adequate learning phase. To date, there are no studies which have investigated gaze agency in any psychiatric population (for indirect evidence, see Lindner et al., 2005). Thus, we have decided to exploit this new paradigm in order to study self-agency in the specific clinical population of OCD checkers (see Belayachi and Van der Linden, 2009, 2010), with the main aim of evaluating if the previous findings on OCD and self-agency could be reproduced using a different effector (i.e., the eye, instead of bodily movements performed through arms or fingers).

## Materials and Methods

### Participants

Twenty-one OCD patients were recruited over 10 months (which fairly correspond to the total experimental period) from consecutive admission to the Department of Clinical Neurosciences at IRCCS San Raffaele Turro in Milan. OCD group was recruited among both outpatients and inpatients. Patients with psychotic spectrum disorders, history of brain injuries and/or of substance use disorder were not included in the sample. All patients who were admitted at the Department in that period and met the inclusion criteria took part to the study. None of the patients asked to exit the study, either before or during the experimental session, indicating that the experimental setting was well tolerated. Twenty-one healthy volunteers (HCs), without psychiatric lifetime diagnoses, were recruited from the general population (i.e., students and experimenters’ acquaintances naive to the purpose of the study and any psychological knowledge) to serve as control group. The two groups were matched for both sex and age (±3 years). Senior psychiatrists made OCD diagnosis according to the Diagnostic and Statistical Manual of Mental Disorders, 4th edition, text revision (DSM-IV-TR) and excluded mental retardation administering the WAIS-R (Wechsler, 1981) only in case of doubt, both to inpatients and outpatients. Only patients with pathological doubt and compulsions to check as their primary symptoms were recruited; moreover, through the Y-BOCS Scale, we assessed also “insight”, which ranges from 0 (i.e., “Excellent”) to 4 (i.e., Absent), “doubt”, which ranges from 0 (i.e., “Absent”) to 4 (i.e., “Severe”), and the severity of the disease, which ranges from 0 to 40 (i.e., 0–7 “Subclinical”, 8–15 “Mild”, 16–23 “Moderate”, 24–31 “Severe” and 32–40 “Extreme”; Goodman et al., 1989). At the testing time, 20 patients were receiving pharmacological treatment: the majority of OCD patients (42.86%) was treated with a combination of medications, most of which (86%) were selective serotonin reuptake inhibitor (SSRI; Table 1). Moreover, 11 inpatients (6 males and 5 females) received both Cognitive-Behavioral Therapy (CBT) and pharmacological treatment. This study was carried out in accordance with the general principles of the Declaration of Helsinki. All subjects gave written informed consent, and the study protocol was approved by the San Raffaele Ethical Committee.

**Table 1:** Demographic and clinical characteristics of study groups.

Demographic and clinical data	OCD (*n* = 21)	HC (*n* = 21)	*p*^1^
*Demographic characteristics*			
Age—mean (SD)	42.29 (15.19)	41.81 (15.91)	0.772
Education—mean (SD)	13.24 (2.49)	15.14 (3.38)	0.030*
Gender—♂/♀	14/7	14/7	1.000^2^
*Clinical characteristics*—mean (SD)			
Duration of illness	24.11 (13.86)	NA	NA
Onset of illness	17.70 (6.72)	NA	NA
BDI-II	16.57 (12.64)	6.21 (7.03)	0.011*
STAI-I	43.86 (12.27)	30.50 (5.75)	0.000**
STAI-II	54.86 (12.19)	37.00 (11.01)	0.000**
LoC	35.75 (11.07)	24.58 (7.84)	0.001**
PADUA F1	27.05 (12.88)	9.16 (11.69)	0.000**
PADUA F2	14.15 (10.60)	5.21 (6.19)	0.001**
PADUA F3	11.50 (7.98)	4.37 (4.87)	0.000**
PADUA F4	3.10 (4.42)	1.11 (1.73)	0.113
PADUA TOT	70.30 (34.87)	24.74 (28.38)	0.000**
VAS PRE	34.17 (23.89)	24.58 (7.84)	0.001**
VAS 1	20.36 (17.17)	12.86 (17.14)	0.002**
VAS 2	30.93 (22.21)	14.43 (19.13)	0.090
VAS 3	29.57 (24.36)	9.81 (14.30)	0.002**
VAS 4	27.36 (26.81)	9.93 (17.39)	0.007**
DY-BOCS Doubt	2.50 (0.83)	NA	NA
DY-BOCS Insight	1.50 (0.69)	NA	NA
DY-BOCS Total	26.35 (8.17)	NA	NA
*Medication at Study Time*	*n* (%)		
Drug free	1 (4.76)		
1 Medication	6 (28.57)		
− Fluoxetine	2		
− Fluvoxamine	2		
− Paroxetine	1		
− Citalopram	1		
2 Medications	9 (42.86)		
− Clomipramine + Sertraline	1		
− Fluvoxamine Maleate + Levothyroxine	1		
− Sodium Valproate + Clomipramine	1		
− Gabapentine + Fluvoxamine Maleate	1		
− Fluvoxamine Maleate + Clomipramine	1		
− Sertraline + Quetiapine	1	
− Clomipramine + Alprazolam	1		
− Fluoxetine + Fluvoxamine	1		
− Clomipramine + Sertraline	1		
>2 Medications	5 (23.81)		
− Clomipramine + Sertraline + Olanzapine + Sodium valproate + Alprazolam	1		
− Fluvoxamine + Pregabalin + Haloperidol	1		
− Clomipramine + Risperidone + Citalopram	1		
− Sertraline + Fluoxetine + Risperidone	1		
− Fluoxetine + Fluvoxamine + Alprazolam	1		

BDI-II, Beck Depression Inventory-Second Edition; STAI-I, State-Trait Anxiety Inventory (State); STAI-II, State-Trait Anxiety Inventory (Trait); LoC, Locus of Control Behavior Scale; PADUA F1, PADUA Inventory Factor 1; PADUA F2, PADUA Inventory Factor 2; PADUA F3, PADUA Inventory Factor 3; PADUA F4, PADUA Inventory Factor 4; PADUA TOT, PADUA Inventory Total Score; VAS PRE, Visual Analogue Scale for Anxiety Pre-Test; VAS 1, Visual Analogue Scale for Anxiety post session 1; VAS 2, Visual Analogue Scale for Anxiety post session 2; VAS 3, Visual Analogue Scale for Anxiety post session 3; VAS 4, Visual Analogue Scale for Anxiety post session 4. ^1^Mann-Whitney U Test. ^2^χ^2^test (χ^2^ < 0.001). *p < 0.05, **p < 0.01.

### Clinical Assessment

The following questionnaires were administered in order to evaluate the clinical variables of interest in both samples: (1) *The State-Trait Anxiety Inventory (STAI-Y)* is commonly used to measure trait and state anxiety (Spielberger et al., 1970). Y form is the most widespread version and comprises 20 items for assessing trait anxiety (e.g., “I worry too much over something that really doesn’t matter”) and 20 for state anxiety (e.g., “I am tense”, “I am worried” or “I feel calm”). All items are rated on a 4-point scale, from “Almost Never” to “Almost Always”: higher scores indicate greater anxiety; (2) *Visual Analogue Scale for Anxiety (VAS-A)* is another common tool exploited to assess contingent anxiety, as it is immediately administered before and/or after a task (Hornblow and Kidson, 1976). A total of 5 VAS is collected for each subject: indeed, one VAS is administered immediately before starting *Discovery* *Task* (i.e., VAS PRE), while the others are administered immediately after each condition (i.e., VAS 1 after Condition 1, VAS 2 after Condition 2, VAS 3 after Condition 3 and VAS 4 after Condition 4). Subjects are required to mark with a bar line their level of anxiety on a continuous line which ranges from 0 (i.e., “No anxiety”) to 100 i.e., “The higher level of anxiety you can imagine”; (3) *Beck Depression Inventory—II (BDI-II)* is a self-report questionnaire which aims to evaluate depressive symptoms severity in adult and adolescent patients (Beck et al., 1996). It consists of 21 items with four response options, ranging from 0 (i.e., “Not Present”) to 3 (i.e., “Severe”). It provides a global score and the higher the score, the more severe the symptoms; (4) *Padua Inventory* is a self-report questionnaire, which consists of 60 items. It describes the most common obsessional and compulsive behaviors related to OCD (Sanavio, 1988). Subjects are required to mark their agreement on a 5-points scale, which ranges from 0 (i.e., “Strongly Disagree”) to 4 (i.e., “Strongly Agree”). The results are divided into five scores: a global score and 4 factors score (i.e., Factor 1—“Impaired control of mental activities”; Factor 2—“Becoming contaminated”; Factor 3—“Checking behaviors”; Factor 4—“Urges and worries of losing control over motor behaviors”). Higher scores mean more severe symptoms; (6) *Locus of Control of Behavior (LoC)* is a self-report questionnaire used to assess the different types of causal attribution people make in their everyday life (Craig et al., 1984). It consists of 17 items (e.g., “I can anticipate difficulties and take action to avoid them” and “My mistakes and problems are my responsibility to deal with”), with a 6-scale of agreement, which ranges from 0 (i.e., “Strongly Disagree”) to 5 (i.e., “Strongly Agree”). All questionnaires were administered immediately before or after the experiment, with the exception of the VAS-A, which was provided always just before the beginning and after every session of the first task (see Table 1 for details regarding the participants’ clinical characteristics obtained through the questionnaires).

### Stimuli and Tasks

We used the same stimuli and tasks described in our previous work (Gregori Grgič et al., 2016). In the following we briefly describe them, but we refer to the original article for details.

#### Discovery Task

Participants were seated in a mildly darkened room, with the head leaning on a chin rest, in front of a computer screen (Asus, LCD, 19″, framerate: 60 Hz, viewing distance: 57 cm). The visual stimulus was made up of gray balls (diameter 2.6 deg) that entered the display sequentially from the bottom left corner of the screen. The balls moved linearly in random directions at constant velocity (5 deg/s), colliding with each other and with the display’s borders, changing their movement energy after each rebound. The number of balls decreased progressively passing from the first to the sixth trial: 30, 15, 10, 5, 2, 1. In the 7th trial, two stationary balls were displayed at 5 deg to the left and the right of the screen center. Each trial lasted 20 s, during which participants also heard through earphones a sequence of beeps, with various inter-beep timing (see below). Participants had to guess the cause of the beeps. At the end of each trial observers reported verbally their supposition about the possible cause of the beeps and rated their confidence (range 1–5, where 1 means null confidence and 5 the highest confidence). When participants answered “I do not know”, a Confidence Rating (CR) of 0 was assigned. The Discovery Task consisted of four conditions. In the Saccades condition the beeps were caused by observer’s saccades (one saccade—one beep). In this condition, an 8th trial was added, identical to the 7th trial, but preceded by a pre-recorded vocal hint “Pay attention to your eyes”. In the Inflating condition participants heard a beep whenever a new ball entered the screen. In the Hemifield condition, when the observer’s gaze pointed to the right hemifield a sequence of beeps was generated (at a constant rate of 4 Hz), while no beeps were generated when observers’ gaze was directed to the left hemifield. In the “Motion” condition the beeps were generated at a rate directly proportional to the instantaneous average velocity of the balls (range: 0.67–4.76 Hz). The four conditions were administered in the following order: “Inflating → Hemifield → Motion → Saccades”, that is, alternating the presentation of an external (Inflating and Motion conditions) and an internal (Hemifield and Saccades conditions) beeps’ cause. After each condition, the experimenter revealed the origin of the beeps, regardless of whether or not participants had already reported the true cause. In this way, all participants started the next condition with the same amount of information. The Discovery Task lasts about 30 min.

The main variables of interest were:

1. **Performance Index (PI)—**Given the binary nature of participants’ responses (i.e., correct or wrong), for each subject and for each condition, the PI was calculated as the ratio between the number of correct responses and the number of trials of the condition of interest. Under the assumption of a monotonic increase as a function of trial number, the PI increased the sooner the observer discovered the correct origin of the beeps;
2. **Confidence Ratings (CRs)—**At the end of each trial, participants gave their rate of certainty about their guessing, which ranged from 1 (i.e., “Not sure at all”) to 5 (i.e., “Absolutely sure”). 0 was given by the experimenter every time participants were not able to give any answer;
3. **Attributional Style—**The four experimental conditions implemented either an external beeps’ cause (Inflating and Motion) or an internal cause (Hemifield and Saccades). Thus, we decided to evaluate the frequency of “External”, “Internal” and “Doubt” responses in the two groups, whether correct or not. An “External” response was qualitatively defined as a “response that does not refer to the subject” (e.g., “the cause of the beeps is the movement of the bubbles!”), an “Internal” response was defined as a “response that refers to the subject” (e.g., “the beeps are associated to my eye movements!”) and we evaluated as a “Doubt” response each trial in which the subject gave “I do not know” as a response. Subjects’ responses were classified by two independent judges and the performance was quantified by measuring the frequency of each type of response;
4. **Erroneous repetitions—**We used the term erroneous repetitions to indicate a response that was already excluded as a possible response (i.e., attributing the same cause implied in a previous experimental condition—subjects were told in advance that this could never occur). As a consequence, a response could count as an erroneous repetition only starting from Condition 2 (Hemifield). Erroneous repetitions were exploited as a measure of cognitive inflexibility.

#### Detection Task

This task was administered always after the Discovery Task, as subjects had to be naïve in the Discovery task. The Detection task consisted of 40 trials, each lasting 10 s. Visual and auditory stimuli were basically the same as in the *Discovery Task*, but the visual stimulus comprised always 10 balls. In half of the trials, the beeps were caused by the observer’s saccades. In the other half of the trials, the beeps were presented with the same sequence produced by the observer during a preceding trial, randomly chosen among the N-1 previous trials. These two types of trials (contingent and non-contingent) were administered randomly. At the end of each trial observers had to report whether the beeps were generated by their eyes or not by pressing a key. A response confidence rating (range 1–5) was also given, again with a key-press. The Detection Task lasted about 20 min.

The main outcomes of interest were:

1. **Accuracy—**The proportion of correct responses;
2. **Confidence Ratings (CR)—**At the end of each trial, participants rated their level of certainty about their guessing, which ranged from 1 (i.e., “Not sure at all”) to 5 (i.e., “Absolutely sure”);
3. **Number of “Hits”—**Subjects’ responses were categorized as “Hits” when the beeps were caused by the subject’s current saccades, and his/her response was “Eyes”
4. **Number of “False Alarms”—**Subjects’ responses were categorized as “False Alarms” when the beeps were not caused by the subjects’ current saccades, but his/her response was “Eyes”;
5. **Number of “Correct Rejections”—**Subjects’ responses were categorized as “Correct Rejections” when the beeps were not caused by the subjects’ current saccades, and his/her response was “Other”;
6. **Number of “Misses”—**Subjects’ responses were categorized as “Misses” when the beeps were caused by the subjects’ current saccades, but his/her response was “Other”;
7. **d-prime (d′)—**It is a non-dimensional measure of the difference between the z-transformed values of “Hits” and “False alarms” rates (Macmillan and Creelman, 2005). The higher d′, the better the subject’s sensitivity;
8. **Bias—**It is a measure of participants’ tendency to prefer one of the two responses, independently of the actual stimulus (Macmillan and Creelman, 2005). A negative value indicated a preference towards ‘Eyes” responses, while a positive Bias value indicated a preference towards “Other” responses;
9. **Oculomotor Parameters—**The oculomotor parameters taken into account were: (a) Fixation duration (s); (b) Saccadic amplitude (deg); (c) Saccadic frequency (number of saccades/s); (d) Time spent in smooth pursuit, e.g., when following with the gaze a ball Motion (s).

#### Eye Tracking and Gaze-Contingency

Two-dimensional eye movements were monocularly recorded through infrared oculometry (Dr. Bouis Oculometer limbus tracker, nominal precision: <0.3 deg, sampling rate: 600 Hz). Before the beginning of each condition in the Discovery Task, and before the beginning of the Detection Task, a 5-points calibration was performed. Gaze contingency was obtained by computing in real time the eye instantaneous tangential velocity. A beep was triggered when eye velocity exceeded the velocity threshold (Discovery Task: 120 deg/s, Detection Task: 60 deg/s; see Gregori Grgič et al., 2016 for details), provided the inter-beep interval was at least 200 ms (“refractory period”, added to prevent possible confounding double beeps). The delay between a beep and a saccade was 50 ms.

### Statistical Analyses

#### Clinical and Demographic Variables

Mann-Whitney U Test was run to evaluate between groups differences in Age, Education, BDI-II, STAI-I, STAI-II, LoC, PADUA F1, PADUA F2, PADUA F3, PADUA F4, PADUA TOT, VAS PRE, VAS 1, VAS 2, VAS 3 and VAS 4.

*χ*^2^ test was used to evaluate differences in gender distribution among the two groups.

#### Discovery Task

Mann-Whitney U Test was run to evaluate between groups differences in PIs and CRs. Wilcoxon test was run to evaluate within groups differences in PIs and CRs. *χ*^2^ test was used to evaluate the proportions of attributional styles.

#### Detection Task

Mann-Whitney U Test was run to evaluate between groups differences in Accuracy, CRs, d′, Bias, Number of “Hits” Responses, Number of “False Alarms” Responses, Number of “Correct Rejections” Responses, Number of “Misses” Responses and the Ocular Parameters. One-sample Wilcoxon signed rank test was run to evaluate if Bias was different from 0 for each group.

#### Correlations Analyses

Spearman Rho coefficient was used to evaluate the association between all the clinical variables and both Discovery and Detection Tasks outcomes.

Normality was checked through Shapiro-Wilk test. For all statistical analyses, we used IBM SPSS 22.0 software.

## Results

### Demographic and Clinical Characteristics

No differences were found in both age and gender between the two groups, but data highlighted a significant difference in education (Table 1). Moreover, significant differences for BDI-II, STAI-I, STAI-II, PADUA Factor 1, PADUA Factor 2, PADUA Factor 3, PADUA TOT, LoC, VAS PRE, VAS 2, VAS 3 and VAS 4 were found, with higher scores totalized by OCD subjects in all these variables (Table 1).

### Discovery Task

#### Performance Index and Confidence Ratings

**Figure 1:**
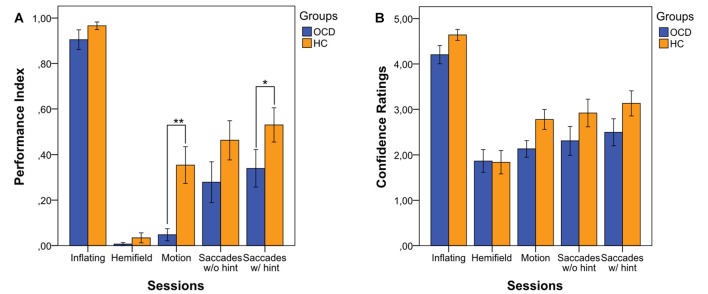
Performance Index (PI) **(A)** and Confidence Ratings (CRs) **(B)** in the four experimental conditions, in both patients obsessive-compulsive disorder (OCD) and healthy controls (HCs). The index values indicate the means of correct responses in each condition for each group. Bars represent means ±1 SE. * Indicates *p* < 0.05 and ** indicates *p* < 0.01 (only the significance of the between comparisons is shown).

Our first interest was to assess whether OCD patients’ performance in the four conditions was different from that of HCs. By analyzing the various experimental conditions, a significant difference between the two groups emerged in the Motion condition (i.e., Condition 3 PI, OCD: *median* = 0.00; IQR = 0.00; HC: *median* = 0.29; IQR = 0.71; *p* = 0.002) and, importantly, in the Saccades condition when considering the 8th trial preceded by the hint (i.e., Condition 4 PI, OCD: *median* = 0.13; IQR = 0.56; HC: *median* = 0.50; IQR = 0.75; *p* = 0.045; Figure 1A). In Condition 1 and Condition 2 (Inflating and Hemifield) performance was almost at ceiling and floor respectively in both groups, which suggests that all subjects found the Inflating condition quite simple and Hemifield quite difficult to solve. Furthermore, OCD patients’ PI was significantly smaller in Motion than in Saccades condition (both without the hint: *p* = 0.020, and with the hint: *p* = 0.001), while for HCs’ this held true only for the Saccades condition with the hint (*p* = 0.016), which indicates a special difficulty for patients in the Motion condition.

We also evaluated the effect of hint, which turned out to increase the performance in both OCD patients (Condition 4 PI without the hint: *median* = 0.00, IQR = 0.71; Condition 4 PI with the hint: *median* = 0.13, IQR = 0.56; *p* = 0.002) and HCs (Condition 4 PI without the hint: *median* = 0.43, IQR = 0.86; Condition 4 PI with the hint: *median* = 0.50, IQR = 0.75; *p* < 0.001).

Moreover, we were interested in understanding whether the participants’ subjective confidence in their own responses differed between the two groups. However, CR showed no statistically significant differences between the two groups in any condition (Figure 1B).

#### Attributional Style

**Figure 2:**
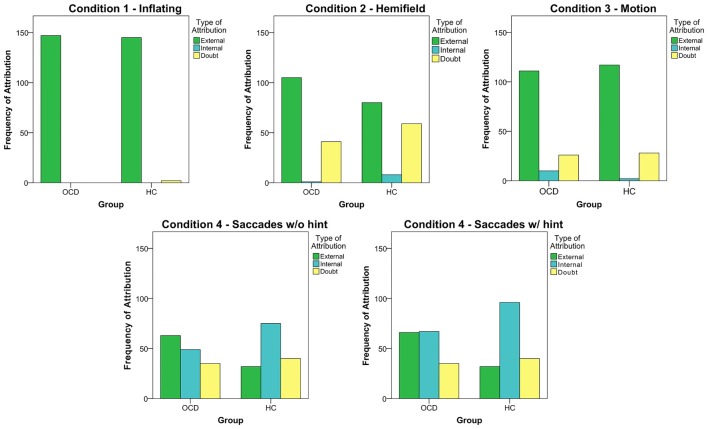
The five graphs represent the frequency of the different type of attribution in each session (the 4th condition, Saccades, was analyzed both considering the 8th trial and without it). Data from all subjects in each group were pooled together.

As a next step, we sought to deepen our analyses by evaluating the type of responses given by subjects. Indeed, as the beep causes could theoretically be linked to an external or internal source, our focus was to investigate what we have called the “Attributional Style” of our subjects: are OCD more prone to attribute to themselves or to external causes the origin of the beeps than HC participants? In general, both groups tended to attribute to external causes the origin of the beeps, as shown by the clear predominance of external attributions in Conditions 1–3 (Figure 2). Condition 3, indeed, is an external condition, but despite the high number of external attributions, subjects showed a low PI, suggesting that this pattern may therefore depend more on task difficulty than on attributional style. However, in these three experimental conditions patients and controls did not differ in terms of attributional style. A difference between OCD and HC emerged in the Saccades condition, as external attribution predominated in OCD patients (External: OCD = 63, HC = 32; Internal: OCD = 49, HC = 75; *χ*^2^ = 15.901; *p* < 0.001), even after the hint was given (External: OCD = 66, HC = 32; Internal: OCD = 67, HC = 96; *χ*^2^ = 17.289; *p* < 0.001).

#### Cognitive (in)flexibility: Erroneous Repetitions (Perseverations?)

**Figure 3:**
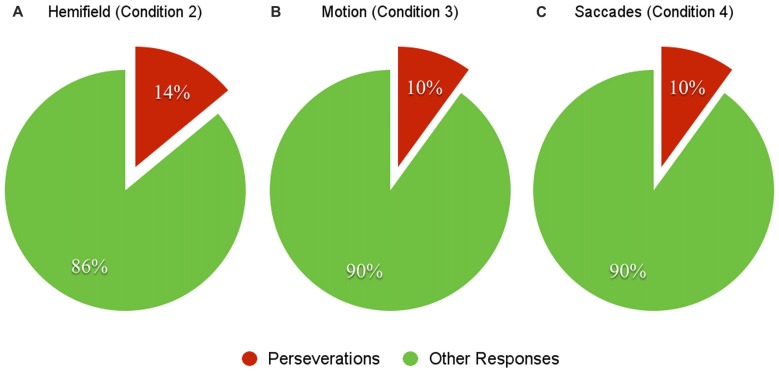
The charts above represent the percentages of erroneous repetitions in condition 2 (Hemifield, **A**), 3 (Motion, **B**) and 4 (Saccades, **C**). Data are relative to OCD patients only, as HC did not produce perseverative responses.

Finally, we have analyzed the number of erroneous repetitions, as they imply a hurdle in modifying the beeps cause theory generated by the subjects. We found that 15 OCD subjects made at least one erroneous repetition, while no HCs made any. Specifically, we evaluated the erroneous repetitions rate in each condition by computing the total number of erroneous repetitions of the condition of interest and the total number of trials, which turned out to be between 10% and 15% (Figure 3). Erroneous repetitions involved more external attributions than internal ones.

#### Correlation Analyses

Concerning demographic data, we have found negative correlations between Age and PI in Condition 1 (*r* = −0.524; *p* = 0.015) and 4 (both without and with the hint: *r* = −0.526; *p* = 0.015 and *r* = −0.568; *p* = 0.007, respectively) in OCD group. Negative correlations between Age and PI were also found in Condition 4 (both without and with the hint: *r* = −0.446; *p* = 0.043 and *r* = 626; *p* = 0.002, respectively) in HC group. Education was not correlated with PIs or CRs in any group: thus, despite it was significantly different among the two groups, it was not associated with any performance variables. Neither Duration nor Onset of illness were correlated with PIs or CRs in the tested conditions.

Regarding clinical data, we have found negative associations between Condition 1 PI and BDI (*r* = −0.539; *p* = 0.012), STAI-I (*r* = −0.466; *p* = 0.033), STAI-II (*r* = −0.507; *p* = 0.019), PADUA F3 (*r* = −0.606; *p* = 0.005) and LoC (*r* = −0.686; *p* = 0.001) in OCD group, while no association has been found between any clinical variables and PIs, or CRs in HC group.

### Detection Task

#### Accuracy, Confidence Ratings, d′ and Bias

At variance with the Discovery Task, in the Detection Task, subjects already knew that their eye movements could or could not be the cause of the beeps. Indeed, in the Detection Task we found a significant difference for detection accuracy, where OCD correct response rate was lower than in HC, but not for CR (Table 2). Moreover, as Detection Task is a two-alternative-forced choice task, we flanked accuracy analyses with d′ and Bias analyses, the former indexing subjects’ sensitivity and the latter indexing subjects’ response preferences. We found significant differences for d′, but not for Bias (Table 2). The lack of Bias difference, coupled with a response tendency which was not significantly higher than zero (OCD: *p* = 0.472; HC: *p* = 0.167), indicates that neither patients’ nor controls’ responses were influenced by tendencies to prefer a given causal attribution.

**Table 2:** Descriptive statistics and *p*-values of accuracy, confidence ratings, d′ and bias.

Variables	OCD	HC	*P*
Accuracy—median (IQR)	0.73 (0.28)	0.82 (0.23)	0.027*
Confidence ratings—median (IQR)	3.04 (1.40)	3.85 (1.97)	0.148
d′—median (IQR)	1.28 (1.75)	2.36 (2.17)	0.021*
Bias—median (IQR)	0.06 (0.51)	0.30 (1.83)	0.196

*p < 0.05.

#### Number of “Hits”, “False Alarms”, “Correct Rejections” and “Misses” Responses

Given the difference in d-prime, we wanted to understand if this dissimilarity was related to False Alarms and/or to Hits responses. We found that only False Alarms significantly differed between the two groups: indeed, OCD subjects made more False Alarms responses than HCs (Table 3). Moreover, concerning Accuracy, we have also taken into account possible differences in Correct Rejections and Misses responses, but only Correct Rejections significantly differed between the two groups. That is, OCD patients gave less Correct Rejections and more False Alarms responses than HCs ones (Table 3).

**Table 3:** Descriptive statistics and *p*-values of “Hits”, “False Alarms”, “Correct Rejections” and “Misses” responses.

Variables	OCD	HC	*P*
Hits—median (IQR)	16.00 (8.00)	17.00 (6.00)	0.260
False alarms—median (IQR)	5.00 (4.00)	2.00 (4.50)	0.014*
Correct rejections—median (IQR)	16.00 (4.00)	18.00 (4.50)	0.014*
Misses—median (IQR)	4.00 (8.00)	3.00 (6.00)	0.260

*p < 0.05.

In addition, CRs for all these types of responses were taken into account. Significant differences were found for Hits (*t* = −2.794, gl = 37.26, *p* = 0.008), and Correct Rejections (*t* = −2.824, gl = 39.31, *p* = 0.007) between OCDs and HCs, while no differences were found for False Alarms and Misses CRs (Table 4).

**Table 4:** Descriptive statistics and *p*-values of “Hits”, “False Alarms”, “Correct Rejections” and “Misses” responses.

Confidence ratings	OCD			HC			*P*
	*n*	*Median*	*IQR*	*n*	*Median*	*IQR*
Hits	21	3.25	3.90	21	4.30	2.55	0.008**
False alarms	20	2.25	4.00	14	2.90	3.35	0.112
Correct rejections	21	2.85	4.80	21	3.70	3.50	0.007**
Misses	19	2.55	4.45	18	3.33	3.00	0.057

**p < 0.01.

#### Ocular Parameters

Significant differences were found for Fixation Duration in Hits, Correct Rejections and Misses and also for Saccadic Amplitude in False Alarms (Supplementary Table S1). These differences, however, were rather small. However, in false alarm trials, saccadic amplitude (which could in principle affect contingency detection), the difference between patients and controls was about 20% (3.08 vs. 3.93 deg, respectively).

#### Correlation Analyses

Concerning demographic data, no associations were found in any of the two groups.

Regarding clinical data, we have found negative correlations between CRs and both DY-BOCS Insight (*r* = −0.486; *p* = 0.035) and Total (*r* = −0.517; *p* = 0.023) in OCD group. We have also found negative associations between Accuracy and BDI-II (*r* = −0.597; *p* = 0.007), STAI-II (*r* = −0.481; *p* = 0.032) and LoC (*r* = −0.507; *p* = 0.027), and between CRs and BDI-II (*r* = −0.481; *p* = 0.037), STAI-II (*r* = −0.682; *p* = 0.001) and LoC (*r* = −0.605; *p* = 0.006). HC group did not show any significant correlation.

## Discussion

The main interest of our explorative study was to investigate the relationship between the SoA and a specific subtype of obsessive-compulsive patients (OCDs): the checkers. To this end, we have exploited a recently developed experimental paradigm, which assessed a particular form of agency, namely, “gaze-agency”, thus allowing us to assess not only manifest alterations of agency sensitivity (Detection Task), but also more subtle signs of agency changes, such as a different attitude to explore the solution space in causal attribution (Discovery Task; see Gregori Grgič et al., 2016).

The Discovery Task revealed a difficulty of causal attribution in OCD patients. Indeed, we found a tendency to a worse performance in patients—that is, a difficulty in finding the true cause of the beeps, as assessed through the objective PI—in Motion and Saccades **(**with hint) conditions. As far as the Saccades condition is concerned (both with and without hint, where the PI difference between OCDs and HCs was 0.37 and 0.43, respectively), it would seem that, compared to HCs, OCDs have difficulty to orient attention towards themselves and rather attribute externally the cause of the beeps, but we cannot tell whether this is more a matter of cognitive (in)flexibility (here a poor reactivity to the a verbal hint) or of a tendency of patients to neglect themselves as agents (here a poor spontaneous ocular self-monitoring before the hint). On the one hand, the fact that in both HCs and OCDs the hint produced a similar and statistically significant PI increase, together with the results of the Detection experiment (see below), suggest that self-monitoring is at least moderately impaired in these patients. On the other hand, the fact that OCD—but not HC—perseverated in reporting a wrong cause of the beeps even when it was clearly wrong, together with their poor performance in the Motion condition, suggest that cognitive inflexibility is a general tract of these patients, at least with difficult tasks. In short, our results suggest that OCDs have difficulties with causal attribution, probably due to both cognitive inflexibility and less functional gaze agency.

In the Detection Task, the main result was a lower accuracy and a smaller d′ in OCD, with no response Bias, suggesting also a lower gaze agency. Indeed, the structure of the Detection Task rules out explanations based on task difficulty. In general, results in HCs were in line with our previous findings (Gregori Grgič et al., 2016). Interestingly, OCD patients exhibited two different attributional tendencies: in the Discovery Task they seemed more prone to externally attribute the origin of the beeps, whereas in the Detection Task they seemed more prone to refer to themselves, at least as revealed by higher false alarms rate (response Bias was not different in HCs and OCDs). Thus, we hypothesized that Discovery and Detection refer to two different levels of agency process: a cognitive and a perceptual one. This hypothesis would clarify why in healthy subjects the two tasks were in some way associated, as subjects could easily understand that their eyes were in some cases the direct cause of the beeps in Discovery and were also accurate in Detection Task (Gregori Grgič et al., 2016), while this was not the case of OCD patients.

### Patients

The OCD group was sampled among only those patients who showed obsessional doubt and compulsions to check as their main content, evaluated through the Y-BOCS (Goodman et al., 1989). According to Goodman et al. (1989) Y-BOCS total score likely revealed a severe case of illness, with good to fair insight and a moderate to severe pathological doubt. Furthermore, the OCD group was well defined in its clinical features, as it showed mild to moderate depressive symptoms in respect to HCs, whose scores revealed none or minimal depressive symptoms (Beck et al., 1988), higher level of both state and trait anxiety and a more external locus of control. OCD patients and HCs were well matched for both age and gender, despite education significantly differed between the two groups. However, education was not associated with any of the performance variables, neither in Discovery, nor in Detection Task.

### Discovery Task: Agency Beliefs

During Discovery Task, subjects were required to create their own causal theories relying only on various contingencies: indeed, during this task, which was administered first, subjects did not know that their eyes could be the cause of the beeps. From a macroscopic point of view, Condition 1 (Inflating) showed a ceiling effect for both groups. Indeed, this condition seems to be the easiest one to discover, as OCD patients and HCs PIs did not significantly differ and were both nearly maximal. Condition 1 was fairly simple in its structure: every single bubble induced a single beep only when it appeared on the screen, hence beeps disappeared when all bubbles were on the screen. The idea that this condition was quite simple was corroborated by the CRs given by the subjects: indeed, they were nearly 4.5 (the range being 0–5) for both groups and were significantly higher than the other sessions’ CRs. Instead, Condition 2 (Hemifield) was a more puzzling condition, not only because it was the first condition in which the cause of the beeps was internal, but also because it had a specific restriction: the beeps were generated only when subjects moved their eyes in the right half of the screen. In fact, it seemed to be the most difficult condition among all, as OCD patients and HCs PIs did not significantly differ and were both nearly 0 (i.e., at floor). It should also be highlighted that in this condition OCD patients made the higher number of erroneous repetitions (*n* = 21). In Condition 3, discovering the beeps cause was also rather difficult, because it implied more abstract reasoning capacity, as velocity is the mathematical combination of two variables: space and time. In this specific condition, subjects were required to integrate both the visual and the acoustic signals in a complex way. Indeed, the beeps frequency was not the same for all the trials, as it depended on the global bubbles velocity: it decreased over trials until trial 7 in which two bubbles on the screen were still. Interestingly, most of OCD patients’ responses were focused only on one of these two variables: someone reported movement of the bubbles (i.e., from the visual input), other reported the presence of a standardized frequency (i.e., from the acoustic input) as the cause of the beeps. Conversely, HC subjects seemed more capable in using both types of cue in order to find the correct solution, even though their PI did not exceed the value of 0.5: they needed nearly half of the trials in order to discover the real cause of the beeps. The worse performance of OCD patients could be therefore linked to cognitive inflexibility. However, OCD patients and HCs’ CRs did not differ and were both lower than 3, indicating a general uncertainty in giving the responses. Lastly, Condition 4 was the principal condition, as it measured subjects’ capability of understanding that their eye movements were the direct cause of the beeps. To do this, observers must first become aware of their own eye movements, which is not obvious (for example, saccadic suppression hides the associated retinal slip from awareness, Wurtz, 2008, and precise saccade timing is also difficult to perceive, Yarrow et al., 2006a,b). However, having already experienced that their eyes could generate the acoustic signals (i.e., Condition 2) should facilitate gaze agency discovery in Condition 4, which could have been otherwise too difficult, especially for patients. As we previously explained, Condition 4 has been split into two sub-conditions, in order to assess whether the clue given before the 8th trial affected or not the PI. The results are interesting, as OCD patients performed significantly worse than HCs in Condition 4 only when the cue was already given and their CRs did not differ in any of the two sub-conditions. The cue had a significant effect in both groups, although OCDs may have been moderately less reactive to this verbal suggestion, as inferred from the fact that the cue introduced an otherwise absent statistically significant PI difference between OCDs and HCs. This possible reactivity difference could be an effect of cognitive inflexibility in patients, which would not allow them to generate novel theories about the cause of the beeps. Furthermore, only OCD patients made erroneous repetitions throughout the conditions.

Our findings are also supported by clinical evidence: indeed, OCD patients show difficulties in shifting between mental processes and adaptive behavioral responses (Gruner et al., 2016). For what is our concern, cognitive flexibility impairment can be conceptualized in the dual-system theories framework: indeed, these theories assume that actions and choices may be supported by either a goal-oriented or a habitual system (Balleine and Dickinson, 1998). Goal-oriented choices and actions allow flexibility, but require more cognitive resources. Habitual choices and actions are more efficient in familiar situations, but do not permit flexibility when the environment changes (Gruner et al., 2016): in fact, an over-reliance on habitual behavior might be the cause of cognitive and behavioral inflexibility. A similar distinction is made between model-based and model-free strategies for action selection in the computational theories framework (Daw et al., 2011). The term model-based describes learned behaviors that require the construction of an internal representation of the task structure to guide the choice. Thus, model-based and goal-oriented learning can be considered quite overlapping constructs, as they imply the construction of an internal representation. Conversely, the term model-free is used to delineate less flexible, but computationally more efficient strategies, which directly link environmental stimuli to responses, bypassing the internal model of the task: indeed, model-free responses seem similar to habitual ones. Interestingly, Voon et al. (2015) found that OCD patients seem more prone to a model-free learning, exhibiting a bias toward less flexible ways of problem solving. It could be hypothesized that these distinctions between goal-oriented/model-based and habitual/model-free responses can be theoretically transferred to the difference between low-level agents (who are more focused on the procedural aspect of an action) and high-level agents (who are more prone to execute an action guided by their goals and take more in account why they are executing an action, rather than how they are performing it) described by Vallacher and Wegner (1989) in their *Action Identification Theory* (AIT). Indeed, Belayachi and Van der Linden exploited Vallacher and Wegner theory suggesting that OCD patients with checking compulsions can be viewed as low-level agents, who are more focused on the procedural aspect of an action and on external cues to represent their internal model of actions and choices (Belayachi and Van der Linden, 2009, 2010). Furthermore, the authors proposed that checkers could have an impaired self-agency, linking it to the construct of not just right experiences, which refers to the impressions of failure or imperfection that could lead to an incapability to fulfil a sense of task completion or closure, related to action and/or perception (Coles et al., 2003). Following their idea, “Not Just Right Experiences” could found their origin in an altered self-agency, since checkers’ bias in parcelling action flow in elementary units may lead them to perform compulsions in a continuous loop. Thus, our preliminary study suggests that OCD patients characterized by checking compulsions might have an altered gaze agency. Indeed, the core feature that could promote “Not Just Right Experiences” (i.e., the feeling of action imperfection and/or incompleteness) seems to be more related to this sensitivity alteration.

### Detection Task: Agency Feelings

For what concern the agency issue the Detection Task aim was to explicitly address gaze agency, since subjects already knew that they could or could not be the cause of the beeps. We found that OCD were less accurate than HCs, since they tended to more frequently attribute to themselves the origin of the beeps. Indeed, their false alarm rate was significantly higher than in HCs nearly in a quarter of the computer-generated trials OCD patients reported that their eyes have caused the beeps and, as a consequence, their correct rejection rate was lower than HCs’ one. Neither the miss rate, nor the hit rate did significantly differ between the two groups. To date, only Gentsch et al. (2012) have experimentally addressed the relationship between self-agency and OCD. Interestingly, our results are in line with Gentsch et al. (2012) ones, as they have found an altered self-agency at a sensory level (i.e., a lack of N1 suppression during self-generated visual events), while their OCD patients’ explicit judgments of agency depended on learned task contingencies to the same extent as those of HCs. Moreover, in that study, OCD patients even showed a trend for increased agency judgments. In a similar way, Detection Task showed an altered d′ due to more False Alarms executed by OCD patients. However, these results should be evaluated with caution. First accuracy was fairly high in both groups. Second d′ exceeded the value of 0 in both groups, indicating that the number of False Alarms was still less than the number of Hits even in OCD patients. Moreover, correlation analyses showed that the higher the depressive symptoms, the anxiety level and an external locus of control the lower the Accuracy only in OCD patients, while no association was found for HCs. This result could suggest that gaze-agency could be affected by psychopathological features associated with OCD. This response pattern seemed to be related to a sensitivity alteration, given OCD patients’ lower d′ values, suggesting a possible less functional gaze agency. By contrast, Bias was not significantly different between the two groups, corroborating the idea that the accuracy difference could be related more to a perceptual level, rather than to the *a priori* beliefs of these subjects.

Concerning the ocular behavior of the subjects, it should be considered that a vast body of literature had already examined eye movements, suggesting possible alterations in OCD patients, although results are highly controversial (Clementz et al., 1996; Pallanti et al., 1996; Rosenberg et al., 1997; Lencer et al., 2004; van der Wee et al., 2006; Damilou et al., 2016). This lack of consistency is probably due to the heterogeneity of clinical samples (i.e., the majority of the studies did not discriminate among the OCD subtypes), pharmacological treatments, methodological paradigms and eye-tracking devices. Our results suggest that none of the ocular parameters was associated with Accuracy, suggesting that the discrepancies between the two groups in correctly guessing the origin of the beeps were not strictly related to their ocular behavior, although it is possible that the decrease of mean saccadic amplitude in OCDs during false alarm trials might have contributed to increase False Alarms detection (smaller saccades may mean less chances to detect contingencies), in turn decreasing patients’ gaze agency sensitivity (d′).

## Conclusion and Future Directions

We operationalized agency through the concept of “gaze agency”, which is a completely new construct, never tested in a clinical population. Due to its novelty, the present results should be considered as a first step towards a full appraisal of agency troubles in psychiatric populations. One aspect that will deserve further scrutiny in the future is to include in the protocol a measure of difficulty and cognitive load in the Discovery Task, as task difficulty *per se* may interfere with agency-based judgments. Given the importance of testing an ecological scenario in which subjects are required to create their own causal hypotheses relying only on task contingencies, this is a cogent issue.

Another issue that deserves further investigation is relating gaze agency performance to causal learning abilities in OCD patients, within the construct of cognitive flexibility. To this aim, we are implementing our investigation flanking Discovery Task with the Intra/Extradimensional Set Shift (IED; Owen et al., 1991), a commonly used test within the Cambridge Neuropsychological Testing Automated Battery (CANTAB) of the *CANTABeclipse*™ software, which evaluates rule acquisition abilities taking into account set-shifting as an index of cognitive flexibility.

Crucial for interpreting OCDs’ performances in terms of dysfunctional neural mechanisms will be to take into account the pharmacological treatment, as a number of drugs could have played a role, such as antipsychotic, benzodiazepine and SSRI (Reilly et al., 2008).

Finally, this approach based on gaze agency could be fruitfully applied also to other OCD subtypes, such as washers (Bloch et al., 2008), as well as to other clinical populations that are known to show both agency and cognitive impairments, such as schizophrenic patients (Haggard et al., 2003; Jeannerod, 2009; Graham et al., 2014; Garbarini et al., 2016; Robinson et al., 2016). Indeed, we hypothesize that, compared to OCD patients, schizophrenic patients would be more productive in their causal theories, despite their inconsistencies and unreality, probably achieving a worse performance both in the Discovery Task and the Detection Task.

## Author Contributions

All the authors met the authorship criteria. MG and RMM specifically contributed to data collection, data analysis, manuscript writing and editing. CS and RGG specifically contributed to experimental design and manuscript writing. SAC specifically contributed to manuscript writing. MCC specifically contributed to clinical diagnosis.

## Conflict of Interest Statement

The authors declare that the research was conducted in the absence of any commercial or financial relationships that could be construed as a potential conflict of interest. The reviewer RB and handling Editor declared their shared affiliation.
