# Supplementary material for: Who Has Done It? Exploring Gaze Agency in Obsessive-Compulsive Checkers
Source: Front Integr Neurosci. 2017 Dec 22;11:39. doi: 10.3389/fnint.2017.00039 (PMC5743893; doi:10.3389/fnint.2017.00039)
Supplement: Supplementary file 1 [file Table_1.docx]

**Table 1S** Descriptive Statistics and p-values of Ocular Parameters (Fixdur: fixation duration; Saccamlp: saccadic amplitude; Sacfreq: saccadic frequency; Smoothdwell: time spent in smooth pursuit)

|  | HIT | | | | FALSE ALARM | | | | CORRECT REJECTION | | | | MISS | | | |
| --- | --- | --- | --- | --- | --- | --- | --- | --- | --- | --- | --- | --- | --- | --- | --- | --- |
|  | *n* | *Mean* | *SD* | *p* | *n* | *Mean* | *SD* | *p* | *n* | *Mean* | *SD* | *p* | *n* | *Mean* | *SD* | *p* |
| Fixdur OCD | 21 | 154,17 | 45,45 | **0.037*** | 20 | 153,02 | 56,41 | 0.102 | 20 | 163,11 | 54,42 | **0.004**** | 18 | 144,23 | 35,74 | **0.026*** |
| Fixdur HC | 21 | 170,41 | 32,88 |  | 15 | 170,73 | 58,00 |  | 15 | 170,04 | 30,59 |  | 19 | 153,59 | 37,38 |  |
| Saccampl OCD | 21 | 3,18 | 0,61 | 0.074 | 20 | 3,08 | 0,51 | **0.004**** | 20 | 3,38 | 0,67 | 0.063 | 18 | 3,03 | 0,82 | 1.000 |
| Saccampl HC | 21 | 3,42 | 0,65 |  | 15 | 3,93 | 1,26 |  | 15 | 3,55 | 0,79 |  | 19 | 3,06 | 0,78 |  |
| Sacfreq OCD | 21 | 1,85 | 0,54 | 0.314 | 20 | 1,89 | 0,65 | 0.756 | 20 | 1,69 | 0,56 | 0.170 | 18 | 1,89 | 0,57 | 0.326 |
| Sacfreq HC | 21 | 1,69 | 0,47 |  | 15 | 1,93 | 0,68 |  | 15 | 1,66 | 0,43 |  | 19 | 1,87 | 0,41 |  |
| Smoothdwell OCD | 21 | 9,14 | 4,85 | 0.199 | 19 | 10,42 | 6,67 | 0.025 | 20 | 8,20 | 3,91 | 0.105 | 18 | 10,54 | 4,33 | 0.097 |
| Smoothdwell  HC | 21 | 7,04 | 2,24 |  | 15 | 5,86 | 3,87 |  | 21 | 6,88 | 2,36 |  | 19 | 8,11 | 4,19 |  |

* p < 0.05, ** p < 0.01
